# Effects of common haplotypes of the ileal sodium dependent bile acid transporter gene on the development of sporadic and familial colorectal cancer: A case control study

**DOI:** 10.1186/1471-2350-9-70

**Published:** 2008-07-21

**Authors:** Frank Grünhage, Matthias Jungck, Christoph Lamberti, Hildegard Keppeler, Ursula Becker, Hildegard Schulte-Witte, Dominik Plassmann, Nicolaus Friedrichs, Reinhard Buettner, Stefan Aretz, Tilman Sauerbruch, Frank Lammert

**Affiliations:** 1 Department of Internal Medicine I, University Hospital Bonn, University of Bonn, Bonn, Germany; 2 Outpatient Clinics for Gastroenterology and Hepatology, Bonn, Germany; 3 Institute for Pathology, University Hospital Bonn, University of Bonn, Bonn, Germany; 4 Institute of Human Genetics, University of Bonn, Bonn, Germany; 5 Department of Internal Medicine II, Saarland-University-Hospital, Saarland University, Homburg, Germany

## Abstract

**Background:**

The genetics of sporadic and non-syndromic familial colorectal cancer (CRC) is not well defined. However, genetic factors that promote the development of precursor lesions, i.e. adenomas, might also predispose to CRC. Recently, an association of colorectal adenoma with two variants (c.507C>T;p.L169L and c.511G>T;p.A171S) of the ileal sodium dependent bile acid transporter gene (*SLC10A2*) has been reported. Here, we reconstructed haplotypes of the *SLC10A2 *gene locus and tested for association with non-syndromic familial and sporadic CRC compared to 'hyper-normal' controls who displayed no colorectal polyps on screening colonoscopy.

**Methods:**

We included 150 patients with sporadic CRC, 93 patients with familial CRC but exclusion of familial adenomatous polyposis and Lynch's syndrome, and 204 'hyper-normal' controls. Haplotype-tagging *SLC10A2 *gene variants were identified in the Hapmap database and genotyped using PCR-based 5' exonuclease assays with fluorescent dye-labelled probes. Haplotypes were reconstructed using the PHASE algorithm. Association testing was performed with both SNPs and reconstructed haplotypes.

**Results:**

Minor allele frequencies of all *SLC10A2 *polymorphisms are within previously reported ranges, and no deviations from Hardy-Weinberg equilibrium are observed. However, we found no association with any of the *SLC10A2 *haplotypes with sporadic or familial CRC in our samples (all P values > 0.05).

**Conclusion:**

Common variants of the *SLC10A2 *gene are not associated with sporadic or familial CRC. Hence, albeit this gene might be associated with early stages of colorectal neoplasia, it appears not to represent a major risk factor for progression to CRC.

## Background

Colorectal cancer (CRC) is a growing burden on health services throughout Western countries. In contrast to familial CRC syndromes, little is known about the underlying molecular mechanisms in the majority of cases. However, the current paradigm is that CRC is due to the interaction of genetic and environmental factors [1]. The effect of each variable may vary considerably, ranging from monogenic familial cancer syndromes that are predominantly caused by genetic defects to sporadic CRCs, which are probably due to life-long exposure to environmental factors with only minor effects of genetic predisposition.

The genes underlying non-syndromic familial CRC are still unknown, or findings have not been reproducible among different studies. A common strategy in experimental genetics is to increase the power by identification of phenotypic extremes and employing association studies in these cohorts, since genetic effects can accumulate in these settings. We have recently successfully translated this strategy to the human situation [2,3]. For the present study we chose to compare the effect of genetic variants in (i) familial CRC cases (after exclusion of Lynch's syndrome and familial adenomatous polyposis [FAP]) and (ii) sporadic CRC patients without a personal or familial history of CRC to a 'hyper-normal' control group with no signs of adenomas or cancer on screening colonoscopy and no personal or familial history of CRC.

Early clinical studies suggested that differences in bile acid metabolism and faecal bile acid composition between CRC patients and controls may contribute to cancer formation [4,5]. In line with this observation, experimental data showed that bile acids can promote DNA adduct formation, induction of proliferation, inhibition of apoptosis, and promotion of tumor invasion [6]. Bile acid composition is influenced by environmental factors such as food intake as well as the bacterial flora in the gut [7,8]. However, bile acid metabolism is also influenced by a number of transport proteins that mediate uptake, distribution or efflux of bile acids. Genetic variation in the genes encoding these transporters together with environmental factors might contribute to CRC development [9-11]. In the intestine, primary bile acids are reabsorbed by active transport in the terminal ileum [12]. This ileal sodium dependent bile acid transporter (ISBT, synonym: apical sodium dependent bile acid transporter ASBT; gene code: *SLC10A2*) is located in the apical membrane of the enterocyte. Only a minor fraction of primary bile acids reaches the colon where the bacterial flora deconjugates the primary bile acids to secondary bile acids such as deoxycholic acid. Disruption of effective reabsorption diminishes the efficacy of the enterohepatic circulation of bile acids and increases the amount of secondary bile acids in the colon [13]. Of note, single nucleotide polymorphisms (SNPs) in the ileal bile acid transporter (*SLC10A2*) gene have been associated with the risk for development of sporadic colorectal adenoma, a precursor lesion for CRC [14]. It remains unclear whether genetic variation in this gene also contributes to the development of CRC.

A haplotype is defined as a specific combination of variant alleles that are inherited together on a single chromosome. In genetically complex diseases such as CRC, haplotypes might provide more information than the analysis of individual polymorphisms, and in some cases an association of a complex phenotype might even be missed if only single markers are analyzed instead of haplotypes [15]. Our aim now was to test whether common variants of the *SLC10A2 *gene are associated with sporadic and familial CRC, employing a haplotype-based association study.

## Methods

### CRC patients

CRC patients were identified in a prospective study on the incidence of hereditary CRC in the area of Bonn and the district of Rhein-Sieg (Northwestern Germany) with about 1 000 000 inhabitants [16]. Part of the inclusion criteria was a structured interview by an experienced physician documenting any personal or family history of CRC. Patients were included in our study if they fulfilled at least two of the three characteristics: (i) three affected relatives, one of them a first-degree relative of the others; (ii) one member diagnosed with CRC before age 50; (iii) two affected generations. Furthermore, we intended to include only cases and controls with Caucasian background; decision was made on appearance and name. This may have led to some misclassification, since no detailed history of ancestry was recorded. However, studies on determination of descent solely by name show that this method is reliable, at least in European populations [17].

Familial CRC patients showed normal expression of *MLH1 *and *MSH2 *in tumor tissues, and microsatellite instability was excluded in all patients. Microsatellite status and immunohistochemistry for *MLH1 *and *MSH2 *expression were determined by the Institute for Pathology at the University Hospital Bonn, which is one of the reference centres for diagnosis of hereditary non-polyposis colorectal cancer (HNPCC) in Germany. Using this combination, Lynch syndrome that is characterized by the development of CRC, endometrial cancer, and other HNPCC-related cancers and caused by a mutation in one of the mismatch repair genes *MLH1, MSH2, MSH6 *or *PMS2 *[18] can be ruled out sufficiently, even though no specific genetic testing for gene mutations was performed [19]. None of the patients had clinical evidence for FAP or any other hereditary disease with an increased risk for CRC.

The second study group consisted of patients with true sporadic CRC. The sporadic nature of the tumor was determined by lack of evidence for any of the inherited CRC diseases (e.g. no signs of FAP or HNPCC, and no family history of CRC). In sporadic CRC cases, tumor stages and localizations were known in 148 and 149 cases, respectively. In familial cases information on tumor stage and localization was available in 61 and 84 cases, respectively.

Controls were recruited from patients seen in our outpatient clinics or in private practice for colonoscopy. Reasons for colonoscopy were positive faecal occult blood test, abdominal pain, or CRC primary prevention. Probands were only accepted as controls when colonoscopy was normal with no apparent sign of abnormal mucosal growth, except that seven patients with hyperplastic polyps (< 5%) were included. The decision was based on histopathology. Individuals were excluded if they had a personal or a family history of CRC or any other tumor or if they reported more than one closely related family member with any malignancy.

All patients and controls gave written informed consent. The study was approved by the Ethical Committee of the University of Bonn.

### DNA extraction and genotyping

Genomic DNA was isolated from EDTA-anticoagulated blood using the QIAamp protocol (Qiagen, Hilden, Germany). Allelic discrimination was performed using fluorescent dye-labelled reporter assays (*Taqman*) with predesigned probes (Applied Biosystems, Foster City, USA) for *SLC10A2 *SNPs *rs157266*, *rs183963*, *rs279941*, and *rs1886927 *[20].

### Haplotypes, tagging SNPs and association testing

We used the online resource of the Hapmap project [21] to select SNPs for haplotype construction and association testing. The Hapmap project provides genome-wide genotype information for dense SNP makers in different reference populations. Using this information a linkage disequilibrium map can be reconstructed, and haplotypes as well as haplotype frequencies can be determined [22]. Genotype data from the Caucasian population was downloaded for the genomic region ranging from position 101,393,000 bp to 101,420,000 bp on chromosome 13, which harbours the *SLC10A2 *gene (based on Hapmap data release 16c.1). The haplotype block structure of the *SLC10A2 *gene was determined with Haploview (release 3.32), using the confidence interval method by Gabriel et al. [23], based on SNPs with minor allele frequencies of > 5%. Within a block some markers carry redundant information. Hence, to fully cover the genetic information of a block only a subfraction of markers, so called tagging SNPs, are sufficient to describe the whole haplotype diversity [15]. Tagging SNPs were determined for the block that also harboured the previously associated SNPs using the Tagger program, as implemented in Haploview. The tagging SNPs were then genotyped in our study populations.

### Association tests for single markers

The software package developed by Strom and Wienker [24] employed for association testing for alleles and genotypes in familial CRC, CRC and 'hyper-normal' controls. Genotype distributions were tested for consistency with Hardy-Weinberg equilibrium using Fisher's exact test. Allele and genotype frequencies were compared using χ^2 ^tests, and Armitage's trend test was calculated to compare the distributions of all genotypes between cases and controls in 2 × 3 tables.

### Haplotype reconstruction in cases and controls

Genotype information from our three study populations was reconstructed using the PHASE algorithm (version 2.2) that is based on Bayesian inference. To test for significant differences in haplotype distributions between familial CRC patients, CRC patients and 'hyper-normal' controls, permutation tests were performed, as implemented in PHASE. The permutation test checks the null hypothesis that case and control haplotypes are a random sample from a single set of haplotype frequencies versus the alternative that cases are more similar to each other than to controls. Haplotypes of cases and controls were permuted 10,000 times, which generates empirical p-values at the 0.05 level while controlling for multiple testing.

### Power estimates for association tests

**Figure 1:**
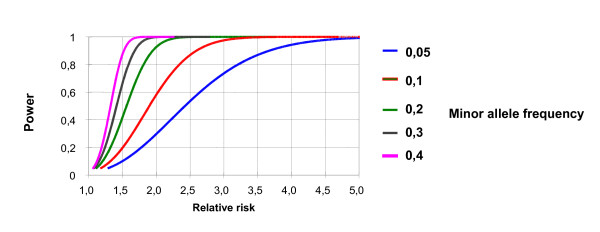
Power estimates for association tests. The figure shows the power (y-axis) of association studies for a range of different minor allele frequencies (0.05–0.40) and varying relative risks (x-axis). The figure demonstrates that power estimates are robust over a wide range of minor allele frequencies that can be expected for the *SLC10A2 *variants.

Power calculations were performed using the P & S power and sample size program [25]. The study population was designed to detect a 2-fold increased relative risk for any marker (SNP or haplotype) with a power of 80% based on a frequency of the risk allele of ≥ 0.1 and a significance level of 0.05. Figure 1 illustrates that the power estimates proved to be robust over a wide range of marker frequencies.

## Results

A total number of 93 familial CRC, 150 sporadic CRC patients and 204 'hyper-normal' controls were included. Table 1 summarizes their clinical characteristics. 'Hyper-normal' controls were age-matched to sporadic CRC patients to reduce the possibility of inclusion of patients who might develop colorectal adenomas or CRC in later life. We found a slight over-representation of left sided tumors in familial CRC patients; in addition, these patients presented with more advanced tumor stages than sporadic CRC patients (Table 1).

**Table 1:** Clinical characteristics of familial CRC, sporadic CRC patients and 'hyper-normal' controls

	**Familial CRC ** **(n = 93)**	**Sporadic CRC ** **(n = 150)**	**'Hyper-normal' controls** **(n = 204)**
**Age**	**55 ± 11**	**66 ± 8**	**62 ± 7**

**Males: females**	**51: 42**	**81: 69**	**91: 113**

**UICCC stage**	**(n = 61)**	**(n = 147)**	
I	13 (21.0%)	34 (23.1%)	
II	9 (14.5%)	52 (35.4%)	
III	26 (41.9%)	48 (32.7%)	
IV	13 (22.6%)	13 (8.8%)	

**Localisation**	**(n = 67)**	**(n = 144)**	
Right Colon	17 (25.4%)	35 (28.9%)	
Left Colon	50 (74.6%)	109 (71.1%)	

### Association tests with single SLC10A2 variants

Data mining from the Hapmap project led to a selection of six SNPs in the *SLC10A2 *gene that define a single haplotype block. Haplotype blocks refer to sites of closely located SNPs, which are inherited together in blocks. Regions corresponding to blocks have a few common haplotypes that are present on a large proportion of chromosomes in the population under study. The strategy is to identify the minimal subset of SNPs (so called tagging SNPs) that characterize the most common haplotypes. Four out of six SNPs were identified as tagging SNPs that are sufficient to cover the whole haplotype diversity without loss of genetic information in this block. Table 2 and 3 summarize the allele and genotype frequencies of the four *SLC10A2 *tagging SNPs. No significant differences in allele or genotype frequencies were observed between patients and controls.

**Table 2:** *SLC10A2 *tagging SNPs and minor allele frequencies

**Tagging SNP**	**Variant**	**Minor allele frequency**			**P values**
			
		**Familial CRC**	**Sporadic CRC**	**'Hyper-normal' ** **controls**	
*rs279941*	G>T	0.14	0.10	0.13	^# ^p = 0.78 * p = 0.27
*rs183963*	A>T	0.35	0.37	0.37	^# ^p = 0.54 * p = 0.91
*rs157266*	G>A	0.13	0.11	0.14	^# ^p = 0.86 * p = 0.20
*rs1886927*	G>T	0.09	0.08	0.09	^# ^p = 0.98 * p = 0.67

^# ^Familial CRC patients vs. 'hyper-normal' controls

* Sporadic CRC patients vs. 'hyper-normal' controls

**Table 3:** Genotype frequencies of *SLC10A2 *tagging SNPs

**Tagging SNP**	**Variant**	**Genotype frequency**			**Armitage trend ** **test**
			
		**Familial CRC**	**Sporadic CRC**	**'Hyper-normal' ** **controls**	
*rs279941*	GG	0.74	0.82	0.76	*p = 0.78
	GT	0.25	0.17	0.22	^#^p = 0.27
	TT	0.01	0.01	0.02	
*rs183963*	AA	0.46	0.39	0.43	*p = 0.56
	AT	0.39	0.49	0.41	^#^p = 0.97
	TT	0.15	0.12	0.15	
*rs157266*	GG	0.74	0.80	0.76	*p = 0.87
	GA	0.25	0.19	0.19	^#^p = 0.23
	GT	0.01	0.02	0.05	
*rs1886927*	GG	0.81	0.84	0.82	*p = 0.98
	GT	0.19	0.15	0.17	^#^p = 0.67
	TT	0.00	0.01	0.01	

* comparison of genotype frequencies between familial CRC and hypernormal controls

# comparison of genotype frequencies between sporadic CRC and hypernormal controls

### Haplotype reconstruction and association tests with common SLC10A2 haplotypes

Four common *SLC10A2 *haplotypes with a frequency > 3% were observed in the Caucasian population of the Hapmap project. Table 4 shows that in line with this observation, we found similar distributions and frequencies of haplotypes in all three groups of patients. However, no significant differences in haplotype distribution were detected between patients and controls. Of note, according to the Hapmap data, the SNPs in codon 169 and 171, which were previously described to be associated with the development of sporadic colorectal adenomas, were in complete linkage disequilibrium with the tagging SNP *rs183963*, as indicated by Lewontin's D' = 1.00. However, *rs183963 *was neither associated with sporadic nor with familial CRC (Tables 2 and 3).

**Table 4:** *SLC10A2 *haplotype frequencies in patients and controls

**Haploptype**	***rs279941***	***rs183963***	***rs157266***	***rs1886927***	**Haplotype frequency**				
					
					**Hapmap project (Caucasians)**	**Familial CRC**	**Sporadic CRC**	**'Hyper-normal' controls**	**Total study population**
***SLC10A2_1***	G	A	G	G	0.48	0.52	0.53	0.50	0.52
***SLC10A2_2***	G	T	A	A	0.32	0.34	0.35	0.35	0.35
***SLC10A2_3***	T	A	A	T	0.13	0.09	0.07	0.08	0.08
***SLC10A2_4***	T	A	A	G	0.04	0.04	0.03	0.04	0.04

## Discussion

Clinical and experimental data suggest a role of secondary bile acids as promoters of colorectal tumors [26,27]. In contrast, water soluble bile acids may counteract, as they induce apoptosis and lower enterocyte proliferation rates in experimental tumor models and in vivo [28,29]. Disturbance of the enterohepatic circulation, e.g. by impaired re-absorption of primary bile acids in the ileum due to genetic variants of the *SLC10A2 *gene, may lead to spillage of primary bile acids in the colon, where they are transformed into secondary bile acids by the endoluminal bacterial flora. In line with this concept, a recent study found an association of a specific mutation (c.507C>T;p.L169L) in the *SLC10A2 *gene and colorectal adenoma development [14]. However, no direct association was found with a nearby non-synonymous mutation (*rs188096*; c.511G>T;p.A171S), indicating that other variants in linkage disequilibrium to the associated locus may contribute to the effect. Although this study [14] found an association with colorectal adenomas, a CRC precursor lesion, the role of the observed association for the development of CRC remains unknown. The study of Wang et al. [14] also observed that a specific genotype combination of the silent mutation and the non-synonymous mutation is associated with colorectal adenoma development. Functional data for the two SNPs studied by Wang et al. are sparse. However, Oelkers et al. performed functional studies for c.511G>T;p.A171S in transfected COS cells but no effect on taurocholate uptake was noticed [14]. In our study, we reconstructed haplotypes of the locus covering the SNPs previously studied by Wang et al. and tested single SNPs as well as complete haplotypes in this block for association with familial and sporadic CRC [14].

By selecting tagging SNPs that covered a whole linkage disequilibrium block we should have been able to detect association with all common variants in linkage disequilibrium with the locus tested by Wang et al. [14]. Our study was sufficiently powered (see *Methods *and Figure 1), and we aimed to further increase the power of our analysis by testing phenotypic extremes (e.g. 'hyper-normal' controls and familial CRC patients), which is a common concept in experimental genetics [20,30] and has been successfully applied by us in previous studies in our cohort of familial CRC patients [2,3]. However, we could not detect such an association with any of the single markers or haplotypes. Hence, variation in the *SLC10A2 *gene appears to be associated with colorectal adenoma, but not with progression to colorectal carcinoma [14]. Our study is limited by a relatively small number of patients with familial CRC. In addition, our clinical information about the included cases is limited and information on life style such as preference of certain diets (e.g. high fiber vs. high fat/meat) and the presence or absence of cholecystectomy which may change bile acid turn over is totally lacking. Thus, we may have missed very small effects of *SLC10A2 *variants on CRC formation due to a type II error and we were unable to study genotype-phenotype interaction with environmental factors that may possibly influence bile salt metabolism.

## Conclusion

In conclusion, development of sporadic and familial CRC is not associated with common haplotypes of the genomic region encompassing the *SLC10A2 *gene previously implicated in the development of colorectal adenomas. However, larger studies may be needed for the detection of very small effects of genetic variants in the *SLC10A2 *gene on CRC development.

## Abbreviations

ASBT: Apical sodium dependent bile acid transporter; CRC: Colorectal cancer; DNA: Deoxyribonucleic acid; FAP: Familial adenomatous polyposis; ISBT: Ileal sodium dependent bile acid transporter; HNPCC: Hereditary non-polyposis colorectal cancer; MLH1: MutL homolog 1, MSH2: MutS homolog 2; SNP: single nucleotide polymorphism.

## Competing interests

The authors declare that they have no competing interests.

## Authors' contributions

FG conceived of the study, performed the statistical analysis and wrote the manuscript. MJ was responsible for data collection and database set up. CL identified patients with familial and sporadic colorectal cancer that were included in the study. HK was responsible for genotyping. UB was responsible for DNA extraction and database management. HSW and DP collected the hyper-normal controls in their outpatient clinic. NC and RB carried out MLH1 and MSH2 staining as well as microsatellite testing in colorectal cancers. SA was central in selecting patients for the study and gave input as specialist for human genetics. TS enabled the study by providing the infra structure and gave advice for conceptional questions. FL supervised the study and wrote the manuscript together with FG.

All authors read and approved the final manuscript

## Pre-publication history

The pre-publication history for this paper can be accessed here:


